# Bartonella endocarditis and diffuse crescentic proliferative glomerulonephritis with a full-house pattern of immune complex deposition

**DOI:** 10.1186/s12882-022-02811-w

**Published:** 2022-05-12

**Authors:** Shunhua Guo, Neha D. Pottanat, Jeremy L. Herrmann, Marcus S. Schamberger

**Affiliations:** 1 Department of Pathology and Laboratory Medicine, Indiana University School of Medicine, 350 W. 11th street, Indianapolis, IN 46202 USA; 2 Department of Pediatrics, Indiana University School of Medicine, Indianapolis, IN USA; 3 Department of Surgery, Indiana University School of Medicine, Indianapolis, IN USA

**Keywords:** *Bartonella henselae*, Cat-scratch disease, Infective endocarditis, Diffuse crescentic proliferative glomerulonephritis, Full-house immune complex deposition

## Abstract

**Background:**

Bartonella endocarditis is often a diagnostic challenge due to its variable clinical manifestations, especially when it is first presented with involvement of organs other than skin and lymph nodes, such as the kidney.

**Case presentation:**

This was a 13-year-old girl presenting with fever, chest and abdominal pain, acute kidney injury, nephrotic-range proteinuria and low complement levels. Her kidney biopsy showed diffuse crescentic proliferative glomerulonephritis with a full-house pattern of immune complex deposition shown by immunofluorescence, which was initially considered consistent with systemic lupus erythematous-associated glomerulonephritis (lupus nephritis). After extensive workup, Bartonella endocarditis was diagnosed. Antibiotic treatment and valvular replacement surgery were undertaken with subsequent return of kidney function to normal range.

**Conclusion:**

This case demonstrates the importance of considering the full clinical picture when interpreting clinical, laboratory and biopsy findings, because the treatment strategy for infective endocarditis versus lupus nephritis is drastically different.

## Background

Cat scratch disease is caused by infection of gram-negative bacilli *Bartonella henselae* [1, 2]. It occurs most frequently in children under age of 15, transmitted from bites or scratches of domestic or feral cat, particularly kittens. Symptoms can include rash or papule at the site of bite/scratch, low grade fever and enlarged tender lymph nodes. Though often a self-limited disease, more severe and systemic spread of the bacteria can cause rare but serious conditions, including ocular and kidney involvement, encephalitis and endocarditis [3, 4]. Glomerulonephritis and endocarditis are uncommon but life-threatening complications in affected patients. Here we report an unusual case of a 13-year-old girl who developed acute crescentic proliferative glomerulonephritis as one of the presenting symptoms of Bartonella endocarditis. Her kidney biopsy specimen was initially interpreted as compatible with lupus nephritis due to the full house pattern of immune complex deposition in glomeruli, highlighting the difficulty in diagnosing Bartonella endocarditis and its associated kidney complication.

## Case presentation

A 13-year-old girl presented 2 weeks prior to a referring hospital with complaints of cough, nasal congestion, chest and abdominal pain, and a fever up to 39.3 °C. She had a history of bicuspid aortic valve status post valvuloplasty in 2007 and 2016 with residual aortic insufficiency and aortic stenosis with aortic root enlargement. She reported no recreational drug use and no known domestic or feral cat or other animal contact history. Her family history was unremarkable except for an uncle with systemic lupus erythematosus (SLE). She was found to have acute kidney injury (creatinine 2.5 mg/dl) with metabolic acidosis, hypertension, nephrotic-range proteinuria, microscopic hematuria (urine RBC > 100/high power field), hypoalbuminemia, and low serum complement C3 and C4 levels. She underwent kidney biopsy which showed diffuse proliferative crescentic glomerulonephritis with full house pattern immunofluorescence finding, which was considered compatible with lupus nephritis. The suspicion for SLE triggered her transfer to our tertiary care center, where further work up was pursued.

### Physical examination

Temperature 36.7 °C, Blood pressure 114/79 mmHg, heart rate 97/minute, respiration rate 21/minute, oxygen saturation 96%, regular heart rate and rhythm, 2–3/6 systolic ejection murmur best heard at right upper sternal border and 2/4 diastolic murmur at lower sternal border, no wheezes or crackles in lungs, soft, non-tender and non-distended abdomen, and no rashes, petechiae or edema on skin.

### Laboratory results

Laboratory test results were summarized in Table 1. Peripheral blood smear showed normocytic anemia, anisocytosis, rouleaux and a few spherocytes with no schistocytes. Blood cultures (10 times) were negative.

**Table 1:** Laboratory results at presentation and at 1 year of follow up

	Presentation	Follow-up	Reference Range
Hemoglobin (g/dl)	7.7	13	12.0–15.0
WBC (× 10^9^/L)	7.2	8.6	3.6–10.6
Platelet (×109/L)	192	387	150–450
BUN (mg/dl)	64	19	5.0–20.0
Creatinine (mg/dl)	2.5	0.68	0.4–0.9
eGFR(ml/min/1.73m^2^)	27.6	88	≥ 90
Total protein (g/dl)	6	NA	6.7–8.2
Albumin (g/dl)	2.7	4.5	3.5–4.7
LDH (units/L)	365	NA	140–270
ALT (uints/L)	3	NA	7–52
ESR (mm/hr)	32	NA	0–20
CRP (mg/dl)	5.2	NA	≤ 1.0
Complement C3 (mg/dl)	34	NA	65–180
Complement C4 (mg/dl)	10	NA	13–52
Anti-nuclear antibody	< 1:80	NA	< 1:80
Anti-dsDNA antibody (IU/ml)	0.8	NA	0–9.9
Smith antibody	negative	NA	negative
anti-RNP antibodies	negative	NA	negative
cardiolipin antiobody (mPL unit)	negative	NA	0–9.9
ANCA	negative	NA	negative
Anti-MPO (AU/ml)	0	NA	0–19
Anti-PR3 (AU/ml)	0	NA	0–19
Urine protein (mg/dl)	500	100	0–15
Urine protein creatinin ratio	12.8	0.98	< 0.2
Urine RBC (/HPF)	> 100	3–5	0–2
Urine WBC (/HPF)	51–100	0–5	0–5

*Abbreviations*: *ALT* alanine aminotransferease, *ANCA* antineutrophil cytoplasmic antibodies, *Anti-MPO* anti-myeloperoxidase antibodies, *Anti-PR3* anti-proteinase-3 antibodies, *Anti-RNP* antinuclear ribonucleoprotein antibodies, *BUN* blood urea nitrogen, *CRP* C-reactive protein, *ESR* Eerythrocyte sedimentation rate, *eGFR* estimated glomerular filtration rate, *HPF* high power field, *LDH* Lactate dehydrogenase, *RBC* red blood cells, *WBC* white blood cells

### Imaging results

**Fig. 1:**
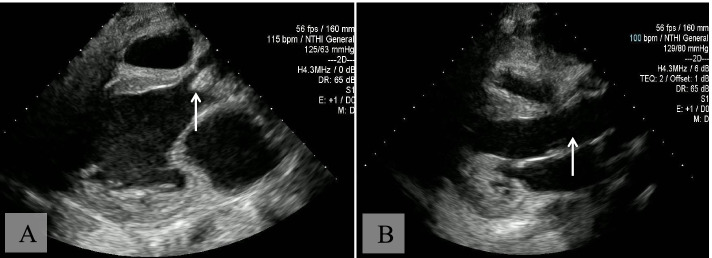
Echocardiogram. **A** Transthoracic echocardiogram, before surgery, two-dimensional, Doppler and color Doppler interrogation, parasternal long axis: Bicuspid and thickened aortic valve with vegetation (arrow), moderate to severe aortic valve regurgitation and mild to moderate aortic stenosis, mildly dilated left atrium and left ventricle with mild to moderate hypertrophy. **B** Transesophageal echocardiogram, after surgery, two-dimensional, Doppler and color Doppler interrogation: There is no left ventricular outflow tract obstruction. On-X mechanical valve shows no vegetation (arrow), with no significant stenosis or regurgitation. Left ventricle is mildly dilated with mild to moderately diminished systolic function

Transthoracic and transesophageal echocardiogram showed bicuspid and thickened aortic valve with thickening of the posterior leaflet of aortic valve, irregular tissue attached to the aortic valve suspicious for vegetation and a pocket area suspicious for a para-valvular abscess (Fig. 1).

### Pathologic finding of kidney biopsy

**Fig. 2:**
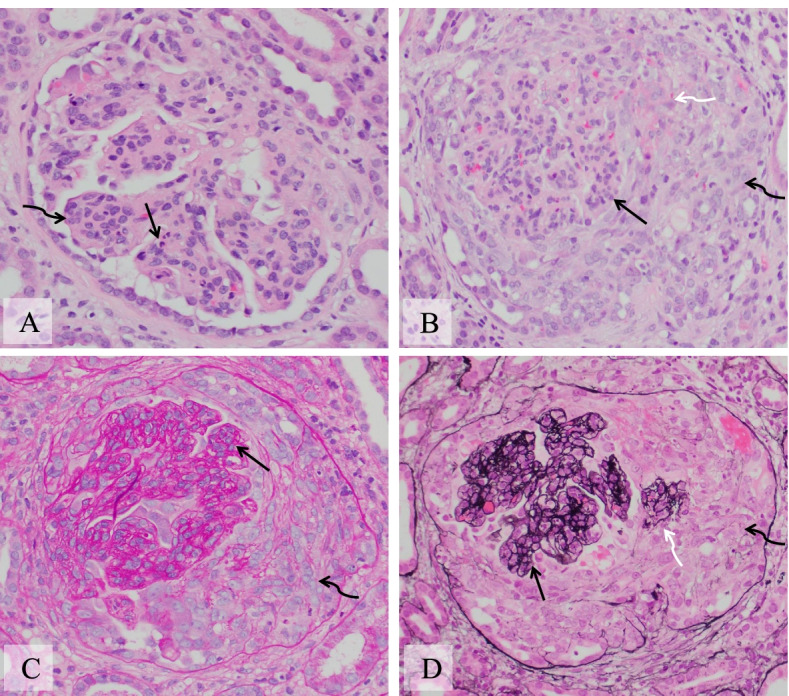
Kidney biopsy, light microscopy (original magnification 400x). **A** (hematoxylin and eosin stain): Glomerulus shows severe global endocapillary hypercellularity (curved arrow). There are neutrophils infiltrating in the capillary lumina (straight arrow). **B** (hematoxylin and eosin stain): A cellular crescent (black curved arrow) surrounds glomerular capillary tufts showing global endocapillary hypercellularity (black straight arrow). There is fibrinoid necrosis (white curved arrow) on the interface of glomerular tuft and crescentic lesion. **C** (Periodic acid Schiff stain): A fibrocellular crescent (curved arrow) surrounding glomerular capillary tufts with global endocapillary hypercellularity (straight arrow). **D** (Jones methenamine silver stain): A cellular crescent (black curved arrow) surrounding glomerular capillary tufts with global endocapillary hypercellularity (black straight arrows). There is rupture of glomerular basement membrane and detachment of a glomerular segment floating in the crescentic area (white curved arrow)

The kidney biopsy material was requested from the referring hospital, including slides of light microscopy with hematoxin-eosin (H&E), periodic acid Schiff (PAS) and Jones methenamine silver stains, and images of direct immunofluorescence microscopy and transmission electron microscopy (EM). Sections of light microcopy showed 33 glomeruli with one globally obsolescent. The remaining glomeruli showed diffuse and severe endocapillary hypercellularity with scattered infiltration of neutrophils in glomerular capillary lumina (Fig. 2). Fourteen glomeruli displayed cellular crescentic lesions, three of which also showed fibrinoid necrosis. Two glomeruli had fibrocellular crescents. There were no spikes or duplication of glomerular basement membranes (GBM) on Jones methenamine silver stain. Mild to focally moderate infiltrates of mononuclear cells were present in interstitium without neutrophils or eosinophils. There was focal interstitial fibrosis (about 10% of cortex). Arteries and arterioles were patent with no vasculitis or thrombi.

**Fig. 3:**
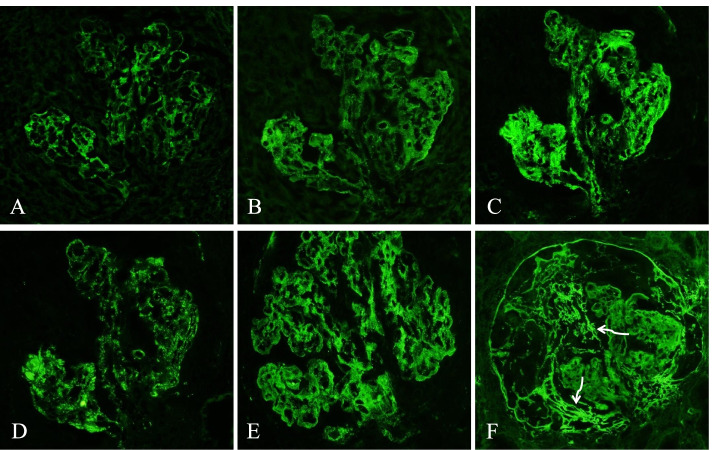
Immunofluorescence microscopy of kidney biopsy (original magnification 400x): Diffuse global granular mesangial and capillary wall deposition of **A** IgG (2+ intensity, on a scale of 0–3+), **B** IgA (2–3+), **C** IgM (3+); **D** C3 (2–3+), **E** C1q (3+) and **F** fibrinogen (3+). Fibrinogen was predominantly detected in the crescentic area (white curved arrows), indicating passage of plasma material to Bowman’s space due to rupture of glomerular basement membrane in the process of fibrinoid necrosis

Immunofluorescence microscopy revealed diffuse granular mesangial and glomerular capillary loop reaction for fluorescein-tagged antibodies to IgG (2+), IgA (2–3+), IgM (3+), C3 (2–3+) and C1q (3+) on a scale of 0–3+. Fibrinogen reaction (3+) was detected in the crescentic area (Fig. 3).

**Fig. 4:**
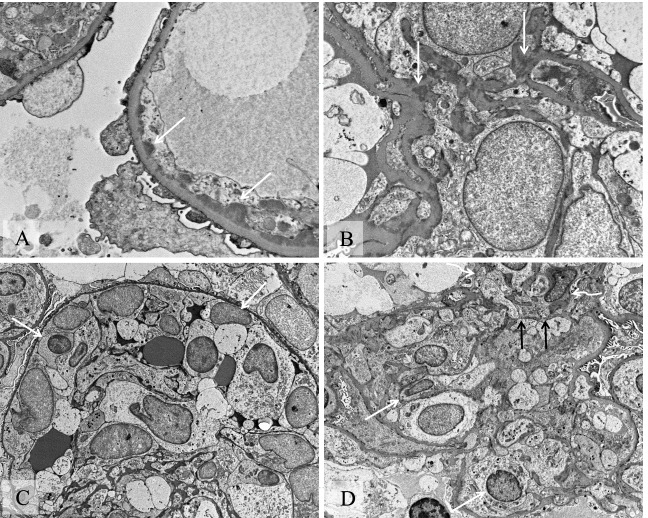
Electron microscopy of a glomerulus: **A** (original magnification 1000x) Multi-foci of subendothelial electron dense deposits (white straight arrows); **B** (original magnification 8000x) Multi-foci of mesangial electron dense deposits (white straight arrows); **C** (original magnification 3000x) Severe endocapillary hypercellularity (white straight arrows). **D** (original magnification 3000x) Severe endocapillary hypercellularity (white straight arrows) and rupture of glomerular basement membrane (black straight arrows) with fibrinoid necrosis and crescentic lesion (white curved arrows) outside of the rupture

Electron microscopy showed frequent glomerular subendothelial and scattered mesangial immune complex-type electron dense deposits (Fig. 4). There were no subepithelial deposits, including no hump-like deposits. There was severe endocapillary hypercellularity. Focal rupture of glomerular basement membrane was observed with adjacent crescent formation. Podocytes display limited effacement of foot processes (about 10%). Glomerular basement membranes showed even contour with normal thickness. Tubuloreticular inclusions were not identified in endothelial cytoplasm.

### Additional laboratory testing and treatment

Multiple additional blood cultures were performed and all resulted negative. Blood sample was sent for Karius next generation sequencing (Redwood City, CA) which revealed Bartonella genomic material. Subsequently, blood Bartonella antibody testing showed anti-*B. henselae* IgG 1:1024 and IgM 1:256; anti-B. *quinata* IgG 1:512 and IgM < 1:16. (reference range for IgG: negative: < 1:64; equivocal 1:64–1:128; positive: ≥ 1:256; for IgM: negative: < 1:16, positive: ≥1:16). Blood was sent to ARUP Laboratory (Salt Lake City, Utah) for Bartonella DNA PCR which detected Bartonella species DNA. Kidney biopsy findings, interpreted in concert with her clinical presentation and new laboratory data, were considered compatible with infection-related acute crescentic proliferative glomerulonephritis, likely associated with clinically diagnosed Bartonella endocarditis. She was started on doxycycline and gentamicin dual agent therapy for 2 weeks.

### Cardiac surgery

After ten additional days of doxycycline therapy, she underwent cardiac surgery. During the operation, it is observed that the ascending aorta was aneurysmal and thin-walled. The left coronary and the noncoronary aortic valve leaflets were heavily calcified and the right coronary leaflet showed a large perforation. There was mass-like material in the right coronary sinus with a pseudoaneurysm through the aortic wall. All the valve tissue was excised in its entirety and the ascending aorta was transected at the juncture. Bentall aortic root replacement was performed with a 23-mm On-X mechanical valve conduit with a 24-mm graft. After the surgery, echocardiogram showed minimal aortic insufficiency, minimal aortic stenosis, improved mitral regurgitation and stenosis, and mildly diminished left ventricular function.

Pathologic examination of the aortic valve tissue revealed multi-foci of necrosis and acute inflammation with calcification, consistent with acute endocarditis. The valve tissue was sent to ARUP Laboratory for Bartonella DNA PCR and showed positive for Bartonella species DNA. Valve tissue culture for both fungal and mycobacteria returned negative.

### Post-operative course and follow-up

The patient received kidney replacement therapy post-operatively for 5 days. Her kidney function gradually improved and she was discharged home 2 weeks after surgery with serum creatinine 0.95 mg/dl. She received doxycycline for another 6 weeks post-surgery. At the one-year fellow up, her serum creatinine was 0.68 mg/dl and urinalysis showed urine protein creatinine ratio 0.98 and RBC 3–5/HPF.

## Discussion and conclusion

Bartonella endocarditis can be a diagnostic challenge due to its variety of clinical manifestations and the difficulty in detection by conventional blood culture [5, 6]. It was first recognized in 1993 when *B. quintana*, *B. henselae* and *B. elizabethae* species were separately reported as the pathogens for endocarditis [7–9]. In a 2005 report, Bartonella accounted for up to 28% cases of culture negative endocarditis [5]. A French reference center reported 106 cases of Bartonella endocarditis diagnosed between 2005 and 2013 [4]. Even with prolonged incubation time, specialized media and special growth conditions, blood cultures are likely to remain negative in most cases [10]. Therefore, Duke criteria for endocarditis, relying heavily on blood culture positivity, are likely to miss a significant proportion of Bartonella endocarditis cases. In contrast, serologic testing for IgG and IgM antibodies to either *B. henselae* or *B. quintana* using microimmunofluorescence techniques can be very helpful for diagnosis. A Bartonella IgG titer of 1:800 is recommended as the threshold for a positive test, offering high sensitivity, specificity, and positive predictive value [4, 11]. PCR detection of Bartonella DNA in blood, serum and valve tissue was reported to be positive in 33, 36 and 91% of cases, respectively [4]. Studies showed that *B. quintana* and *B. henselae* had tropism for endothelial cells and persists in the endothelium, including valvular endothelium [2, 12]. The majority of affected valves were native, but prosthetic valve involvement by Bartonella was also reported and appeared to be more aggressive with valve perforation and rapid development to heart failure [13].

Our patient had a history of valvuloplasty with residual aortic insufficiency and stenosis, which might predispose to invasion by microorganisms. Cardiac surgery was performed after 24 days of appropriate antibiotics treatment, but examination of the valve tissue showed multi-foci of acute inflammation and necrosis with extensive calcification, suggesting that there was still ongoing acute inflammation and necrosis, and the extensive calcification was likely the sequala of severe destruction by infection. Therefore, this finding reiterated that valvular replacement was often necessary to remove the nidus of infection and restore normal valvular function even if appropriate antibiotic treatment could be initiated after the pathogen was identified.

Kidney injury was commonly reported as a complication of infective endocarditis (IE), affecting up to 40–50% of patients, and hence it is included as a minor Duke criterion for the diagnosis of IE [14]. The causes of kidney injury include glomerulonephritis (GN), septic emboli, cortical necrosis, and tubulointerstitial toxicity or allergic reaction to antibiotics. IE caused by many organisms had been associated with glomerulonephritis, such as Staphylococcus (53%), Streptococcus (23%), *Bartonella henselae* (8%), *Coxiella burnetii* (2%), *Cardiobacterium hominis* (2%), Gemella (2%) in one report of 49 cases [15]. The pathogenesis of GN in IE can be divided to two categories. The first is an immune complex-mediated process, which is a prototype of infection-related glomerulonephritis. The second is anti-neutrophil cytoplasmic antibody (ANCA)-associated glomerulonephritis. ANCA positivity was found in 22.7% of gram-positive bacterial endocarditis group and in 60% of Bartonella endocarditis group, with PR3-ANCA antibody found in 40% patients of Bartonella endocarditis group [16, 17]. Other reported kidney manifestations of bartonella infection include renal microabscesses, thrombotic microaniopathy (TMA) and hemophagocytic lymphohistiocytosis (HLH), which were mostly seen in patients with kidney transplant [18, 19]. Immunosuppression in these patients may result in disseminated visceral infection of Bartonella. TMA is likely associated with severe injury of endothelial cells because they are the main niche for Bartonella organisms. The systemic infection in immunocompromised patients may trigger massive release of cytokines that cause extensive activation of macrophages, resulting in HLH [19].

The patient’s glomeruli showed diffuse endocapillary hypercellularity, infiltration of neutrophils in the glomerular capillary lumina, and IgM-dominant deposits in the absence of GBM duplication, all of which are suggestive of infection-related glomerulonephritis. IE-associated glomerulonephritis often shows severe crescentic lesions and fibrinoid necrosis, in addition to the diffuse endocapillary and mesangial proliferation, as seen in this case. Clinically, IE patients also not uncommonly demonstrate decreased serum complement levels, which was also seen in this patient, likely due to systemic activation of complement pathway by antibody-antigen immune complex in the circulation. Even though hump-like subepithelial deposits could be helpful for diagnosis of infection-related glomerulonephritis if detected ultrastructurally, their presence in endocarditis-related diffuse proliferative glomerulonephritis was reported to be only 14% [15].

Even though the full house pattern by immunofluorescence is often seen in lupus nephritis, IgG was weaker than IgM in our patient’s biopsy, but lupus nephritis would more commonly show IgG dominance instead of IgM [20]. In addition, there were no tubuloreticular inclusions in the cytoplasm of endothelial cells by EM to further suggest lupus nephritis. From a pathological perspective, the full house immunofluorescence pattern alone would not be sufficient for diagnosis of lupus nephritis. Instead, a kidney biopsy diagnosis of lupus nephritis should be approached with great caution if SLE is not clinically diagnosed. With the benefit of hindsight, the patient’s diagnosis might be more appropriately reported as “diffuse proliferative glomerulonephritis with extensive crescentic lesions” followed by a comment delineating pertinent light, immunofluorescence and EM findings and discussing differential diagnosis including infection-related glomerulonephritis, lupus nephritis and other idiopathic or secondary immune complex-mediated glomerulonephritides. Specifically, with the clinical history of cardiac valvular disease and echocardiogram results, the possibility of IE-associated glomerulonephritis should be added in the comment and even better conversed directly with the clinician. Again, this case underlined that the biopsy pattern of changes must be interpreted in the context of clinical information so as to point to the correct etiology.

From a clinical perspective, the patient’s multiple SLE serology tests for ANA and anti-dsDNA antibody were negative. Other autoantibodies including Smith and RNP antibodies were also negative. In the new ACR/EULAR criteria, positive ANA was set as the entry criterion for diagnosing SLE [21]. If we disregarded this entry criterion, the patient had fever, pleural effusion, acute glomerulonephritis, and low serum complements, which would add up to 21 points, well above the 10 points required for SLE diagnosis. Therefore, this case substantiates that the ANA entry criterion is important and that the overall clinical picture is critical for avoiding erroneous application of the SLE criteria in clinical practice.

There were reports of so called “renal limited lupus nephritis”, which is still a controversial term [22]. For example, in one report, three of the four cases showed full house immunofluorescence pattern and all four cases showed tubuloreticular inclusions in endothelial cells, but all the patients lacked clinical and serological evidence of SLE (except one case had ANA titer of 1:160) over a mean follow-up period of 3 years [22]. Other reports used the term “non-lupus full-house nephropathy” (NLFHN), which was seen in patients with a variety of pathologic changes and clinical manifestations (Table 2) [22–33]. In one report of 24 patients, only two patients was eventually diagnosed as SLE during follow up of 6–84 months [24]. In another report of 32 patients, none of them were clinically diagnosed as SLE during a median follow up of 20 years [25]. Therefore, it seems that NLFHN is a heterogeneous group of diseases, of which only a small fraction of patients may develop additional features that are diagnostic of SLE. The common feature of full house immune deposits may be caused by deposition of some types of immune complex that can activate both classic and alternative complement pathways. Because there were only rare case reports of full house immune complex deposition in endocarditis-associated proliferative GN, it was unclear what clinical differences were there between IE patients whose GN showed a full house pattern and those who did not [34, 35].

**Table 2:** Literature review: pathologic features and clinical presentations of “non-lupus full-house nephropathy” (NLFHN)

Groups	Pathologic Diagnosis	Cases	Light Microscopy Pattern	Location of Deposits by EM	Clinical Presentation	Literature
	**Idiopathic**					
1	Minimal change	1	Minimal lesion pattern	mes and subendo	normal urinalysis (donor kidney at 0 hour biopsy)	#26
		2		NA	NS	#25
		1		mes	NS	#27
2	Mesangial proliferative GN	1	Mesangial proliferative pattern	NA	SubNS, hematuria	#25
		1		NA	NS and nephritic	#28
		1		mes	SubNS	#24
3	Focal proliferative GN	5	Focal proliferative pattern	2 subepi; 1 TM and subepi; 2 NA	NS; or NS and hematuria	#25
		2		mes, subendo, subepi	NS	#23
4	Diffuse proliferative GN	2	Diffuse proliferative pattern	NA	RPGN, NS, malignant HTN	#25
		1		NA	SubNS, hypertension	#29
		7		NA	NS; or subNS; or NS and nephritic; or RPGN	#28
		10		NA	NS; or nephritic; or NS and nephritic; or CRF	#30
		7		mes, subendo, subepi	NS or subNS, hematuria, renal insufficiency	#23
5	Diffuse proliferative GN with crescents	2	Diffuse proliferative with crescents	mes, subendo, subepi; subepi	RPGN, subNS, hematuria, malignant HTN	#25
		10		NA	NA	#30
		2		mes, subendo, subepi	SubNS and hematuria	#22
		1		mes, subendo, subepi	SubNS, hematuria and renal insufficiency	#23
6	MPGN	1	Membranoproliferative pattern	mes, subendo, subepi	NS	#25
		2		NA	NS and nephritic	#28
		3		mes and subendo	RPGN; chronic renal failure;or NS	#24
		1		NA	NS and nephritic	#29
		12		NA	RPGN; or NS; or NS and nephritic	#30
		1		mes, subendo, subepi	NS, hemturia	#22
		3		mes, subendo, subepi	NS, hematuria	#23
7	MN	1	Membranous pattern	NA	NS, renal insufficiency	#29
		2		NA	1 NS; 1 NS and nephritic	#28
		10		subepi	NS; or subNS	#24
		21		NA	NS; or subNS with nephritic	#30
		4		mes, subendo, subepi	NS or subNS	#23
8	Diffuse proliferative GN with MN	1	Diffuse proliferative and membranous patterns	mes, subendo, subepi	NS, hematuria, renal insufficiency	#22
9	FSGS	2	Segmental chronic lesions	1 mes, subendo, subepi; 1 NA	NS	#25
10	FSGS with TMA	1	Segmental chronic lesions and TMA	NA	NS	#25
11	C1q nephropathy	1	Diffuse proliferative pattern	mes and subendo	NS	#24
		3	Proliferative GN in 1; minima change in 2	NA	NA	#30
12	IgA nephropathy	1	Segmental chronic lesions	NA	SubNS and hematuria	#25
		3	Focal mesangial proliferative pattern	mes	NS; SubNS; or chronic renal failure	#24
		16	Mesangial proliferative pattern	NA	NA	#30
13	IgA nephropathy with crescents	3	Focal or diffuse proliferative pattern with crescents	1 subendo; 2 NA	SubNS, hematuria, RPGN, malignant HTN	#25
		2	Focal or diffuse proliferative pattern with crescents	mes	RPGN	#24
	**Secondary**					
14	Infection-related GN	1	Minimal lesion pattern	mes and subendo	Microalbuminemia, hematuria	#25
		1	Diffuse proliferative pattern	mes, subendo, TM, subepi humps	RPGN	#25
15	Infecious endocarditis-related GN	1	Diffuse proliferative pattern	subepithelial humps	MSSA infectious endocarditis	#24
16	Post-streptocococal GN	2	Diffuse proliferative pattern	subepithelial humps	Nephritic; or NS	#24
17	IgA-dominant infection-related GN	2	Diffuse mesangial proliferative pattern	NA	NA	#30
		1	Diffuse proliferative pattern	subepithelial humps	Nephritic	#24
18	HBV-associated MN	1	Membranous pattern	subepi	NS	#24
		1	Membranous pattern	NA	NS	#30
19	HIV-associated immune complex GN (6 cases with concurrent HCV infection)	6	Focal proliferative pattern	mes, subendo, subepi	NS or subNS, hematuria, renal insufficiency, 1 HCV+	#31
		7	Diffuse proliferative pattern	mes, subendo, subepi	NS or subNS, hematuria, renal insufficiency, 4 HCV+	#31
		1	Membranous pattern	subepi, TM, subendo, mes	NS, HCV+	#31
20	ANCA-associated GN	2	Diffuse proliferative pattern with crescents	NA	RPGN; or subNS and hematuria	#25
		1	Membranoproliferative pattern with crescents	NA	RPGN, HTN crisis, p-ANCA positive	#32
21	Amyloidosis	7	Amyloid protein deposition	NA	NA	#30
22	Cancer-associated MN	3	Membranous, anti-PLA2R negative	1 mes, subepi; 2 subepi	NS; or subNS and hematuria; all with cancer	#25
23	Glomerular disease in IPEX patient	1	Membranous with focal proliferative pattern	mes, subendo, subepi	NS, hematuria	#33

*Abbreviations*: *ANCA* anti-neutrophil cytoplasmic antibody, *EM* electron microscopy, *C1q* complement 1q, *FSGS* focal segmental glomerulosclerosis, *GN* glomerulonephritis, *HBV* hepatitis B virus, *HIV* human immmunodeficiency virus, *HCV* hepatitis C virus, *IgA* immunoglobulin A, *MN* membranous nephropathy, *MPGN* membranoproliferative glomerulonephritis, *MSSA* methicillin-susceptible *Staphylococcus aureus*, *NS* nephrotic syndrome, *IPEX* immunodysregulation, polyendocrinopathy, enteropathy, X-linked, *Mes* mesangial, *NA* not available, *RPGN* rapidly progressive glomerulonephritis, *Subendo* subendothelial, *SubNS* subnephrotic, *Subepi* subepithelial, *TM* transmembrane, *TMA* thrombotic microangiopathy

In conclusion, we present an unusual case of Bartonella endocarditis-associated diffuse crescentic proliferative glomerulonephritis with a full-house pattern of immune complex deposition, which was initially considered consistent with lupus nephritis. The biopsy specimen showed extensive glomerular crescentic lesions but ANCA serology was negative. Antibiotic treatment and valvular replacement surgery were undertaken with subsequent return of kidney function to normal range. This case demonstrated the importance of considering the full clinical picture when interpreting the results of biopsy, laboratory and clinical findings, because the treatment strategy can be drastically different. The recovery of kidney function in this patient with severe crescentic glomerulitis with antibiotics and surgery but without immunosuppression treatments testified for the significance of making distinction in diagnosis between autoimmune and infection-related glomerulonephritis.

## Data Availability

The data of this case report were all from the patient’s electronic medical records. The clinical note, imaging and pathology reports after identifying/confidential patient information is removed can be available upon request.

## Abbreviations

ACR/EULAR: American College of Rheumatology/European League Against Rheumatism
ANA: Anti-nuclear antibody
ANCA: Anti-neutrophil cytoplasmic antibody
DNA: Deoxyribonucleic acid
EM: Electron microscopy
HPF: High power field
GFR: Glomerular filtration rate
GBM: Glomerular basement membrane
GN: Glomerulonephritis
IE: Infective endocarditis
IF: Immunofluorescence
Ig: Immunoglobulin
MPO-ANCA: Myeloperoxidase anti-neutrophil cytoplasmic antibody
PCR: Polymerase chain reaction
PR3-ANCA: Proteinase 3 anti-neutrophil cytoplasmic antibody
SLE: Systemic lupus erythematosus
